# Gendered Experiences of Intravenous Iron Infusion Reactions: A Patient Perspective

**DOI:** 10.1177/23743735251314842

**Published:** 2025-01-28

**Authors:** Roxanna Nasseri Pebdani

**Affiliations:** 1 Centre for disability Research and Policy, Sydney School of Health Sciences, Faculty of Medicine and Health, 4334 The University of Sydney, Sydney, Australia

**Keywords:** hypophosphatemia, iron infusion, women's health, medical bias

## Abstract

Hypophosphatemia following iron infusion is thought to be a relatively rare complication of intravenous iron infusion, though research is beginning to demonstrate that it occurs more often than previously assumed. Still, healthcare professionals are often unaware of this potential complication. This, coupled with the medical bias women can receive in health settings—plus the over-representation of women receiving intravenous iron—means that symptoms of hypophosphatemia may be misinterpreted or worse, misdiagnosed. This article presents an account of a patient experience of hypophosphatemia following intravenous iron. Key points include: healthcare providers should be aware of this potential complication, healthcare providers must be prepared to listen, assess, address, and treat potential hypophosphatemia that occurs after receiving an iron infusion, and physicians should monitor phosphate, particularly in patients who develop fatigue, nausea, bone pain, and other symptoms of hypophosphatemia after receiving an iron infusion. Further research on the prevalence, impact, and duration of hypophosphatemia following iron infusion is needed.

## Introduction to the Issue

My low iron was first diagnosed in 2012, when I lived in Seattle. I had been a vegetarian for a number of years, I was a woman of reproductive age, and I was a runner and a cyclist. So, I began eating meat, cooking with cast iron cookware, and supplementing with oral iron. Over a decade, 2 kids, an international move, a busy job, and 3 half marathons later, I finally found the fatigue, headaches, and weakness too much to manage. So, I spoke with my general physician (GP) and we finally decided on an iron infusion. Yes, there are risks with an infusion—the risk of a permanent ink stain, a small risk of anaphylaxis, but it seemed worth it.

Fortunately, I didn’t have either of those side effects. Instead I ended up with an “iron flu” that lasted about 4 days. Short and manageable. Finally, as I began to feel better, I picked up my energy and went back to normal, ready for the iron elixir to work its magic.

Days later, I was back at the GP—with even worse fatigue, nausea, and an unimaginable headache. I was unable to work—and could barely get myself to my GP's office. A few days after that, I ended up in hospital for the first time. I was diagnosed with hypophosphatemia—clinically low phosphate, an issue I’d never experienced before.

A week later, I had my second hospital visit. There, I was told the symptoms I was experiencing were likely in my head and due to anxiety. Certainly, I was anxious, as I was feeling and looking so unwell a colleague had to walk me 2 blocks to the emergency department. With my partner away for work, I had to make sure I could be healthy and home to pick up my children from school. However, what I was experiencing werealso symptoms of hypophosphatemia—headache, fatigue, bone pain, and a racing heart.

The impacts were ongoing. My previously healthy, though tired and headached, self could no longer work a full day much of the time, even from bed. The days I went into the office had to be short and followed by days of recovery. The fatigue lasted months—through daily phosphate supplements, the aforementioned 2 hospital visits, 5 rounds of blood work, numerous sick days, and countless GP visits. I had headaches so bad I could not move my head. Incredibly low blood pressure. Nausea that just would not pass. I was feeling bone, joint pain, muscle pain— having aches while laying down, not having exerted myself.

I found support in an unlikely place: a Facebook group of people with similar experiences. As a millennial, I’m no stranger to maintaining connections by Facebook, but as an experienced academic, I was in no way prepared to learn that peers experiencing the same symptoms—communicating with one another on a Facebook page—would have what little scientific research that exists on hypophosphatemia postiron infusion at their fingertips.

The 2200 members of this Facebook group came from all over the world. There were chats specific to Australia (where I live, and where I am fortunate enough to have universal healthcare), the United Kingdom, and the United States, and members living in so many more countries—receiving varied forms of care. Group members regularly had to advocate for newer members to request more frequent phosphate measurements after doctors sent people away with the plans to check phosphate again in a few weeks (or not at all). Group members shared research articles, information from their doctors, recommendations for doctors in their area who were knowledgeable about the condition, along with support and care for people who were experiencing a harrowing medical condition—often without sufficient medical support.

All in all, I had it relatively easy, part of which is the immense privilege I have as a PhD educated researcher. I had a general practitioner who respected my voice, listened to me when I brought in information, and worked with me to find solutions (my hospital visit where I was told I was fine but anxious notwithstanding). Not everyone has the same access, information, or voice to advocate for themselves.

Physically, my recovery was relatively swift. I spent a week off work, then a week or 2 unable to work a full day, then I graduated to half days, then started being able to go into the office more regularly. I started again being able to both work and take care of my kids in the same day (a feat initially unattainable due to the fatigue), gradually returning to life, and after a few months, even returning to bike-commuting and eventually even returning to running. Other people in this Facebook group are still unwell—some for over a year, over 2 years, supplementing phosphate every day, but still experiencing fatigue, nausea, bone pain, and other symptoms, all of us whom have one preceding incident—an iron infusion.

## Key Factors for Consideration

There is a small but growing body of evidence that some people experience hypophosphatemia following intravenous injection of ferric carboxymaltose. And yet, somehow, there's a whole group of people who experience this who have no idea that it was possible and who have doctors who are similarly unaware.

There are theories—that some people experience a change in fibroblast growth factor-23 after receiving an iron infusion, leading to hypophosphatemia.^1^ A recent systematic review of 40 studies on intravenous iron infusions in adults found that there is a lack of research on the subject—and what little research exists most likely underestimates how often hypophosphatemia occurs after intravenous iron infusion.^2^ Further, this research often does not track patients beyond 5 to 6 weeks, which limits understanding of the longer-term impacts of the condition.^3^ Given the lack of clinical research, it is unsurprising that many physicians are unaware of these connections or how to treat the condition.^4^

This is likely compounded by other factors, such as historical biases against women seeking treatment for pain^5^ or gender-related misdiagnoses^6^ (you will remember that my physical symptoms were dismissed as anxiety in my second hospital visit). This complicated history of medicine for women coupled with the fact that women are at a higher risk of iron deficiency and therefore more likely to need iron infusions^7^ means that women are more likely to experience this debilitating and perhaps rare side effect, but also more likely to not be listened to when sharing their experiences.

## Recommendations

Given the lack of research and limited clinical knowledge, coupled with the potential for gendered impacts on care, healthcare providers should be doubly prepared to listen, assess, address, and treat potential hypophosphatemia that occurs after receiving an iron infusion. Physicians should monitor phosphate, particularly in patients who develop fatigue, nausea, bone pain, and other symptoms of hypophosphatemia after receiving an iron infusion.^8^ High-quality research should explore the connection between iron infusions and hypophosphatemia: What risk factors make someone more likely to experience this postinfusion? How long does hypophosphatemia typically last after an iron infusion? How can patients be supported, and symptoms be managed during these acute episodes? Are specific formulations of iron infusion are more likely to lead to hypophosphatemia—though there are some answers to this question already, see: Wolf, Chertow, Macdougall, Kaper, Krop and Strauss^9^?

Further, attention should be given to the utility of Facebook groups as a potential source of support, information, and guidance when experiencing lesser-known medical conditions. Researchers may want to make patient-facing literature formatted for these sources, to increase the reach of their results. These groups are typically monitored by people with lived experience of the condition, but perhaps without medical training. Any support the medical community could provide in increasing access to appropriate and accurate information in groups like these could have wide-reaching impacts.

## Conclusion

Hypophosphatemia following iron infusion is a more common occurrence than previously understood, and occurs most often after ferric carboxymaltose infusions.^10^ Further high quality and longitudinal research on hypophosphatemia post iron infusion is needed. Healthcare providers should be aware of this potential complication, which occurs more often than previously thought.
